# The Impact of COVID-19 Pandemic on Long-Term Care Facilities Worldwide: An Overview on International Issues

**DOI:** 10.1155/2020/8870249

**Published:** 2020-11-04

**Authors:** Dana-Claudia Thompson, Madalina-Gabriela Barbu, Cristina Beiu, Liliana Gabriela Popa, Mara Madalina Mihai, Mihai Berteanu, Marius Nicolae Popescu

**Affiliations:** ^1^Fetal Medicine Excellence Research Center, Alessandrescu-Rusescu National Institute for Mother and Child Health, 365 Calea Grivitei street, 010717 Bucharest, Romania; ^2^Department of Dermatology, Elias University Emergency Hospital, 17 Marasti Blvd., 011461 Bucharest, Romania; ^3^Department of Rehabilitation Medicine, Elias University Emergency Hospital, 17 Marasti Blvd., 011461 Bucharest, Romania

## Abstract

The COVID-19 pandemic had a great negative impact on nursing homes, with massive outbreaks being reported in care facilities all over the world, affecting not only the residents but also the care workers and visitors. Due to their advanced age and numerous underlying diseases, the inhabitants of long-term care facilities represent a vulnerable population that should benefit from additional protective measures against contamination. Recently, multiple countries such as France, Spain, Belgium, Canada, and the United States of America reported that an important fraction from the total number of deaths due to the SARS-CoV-2 infection emerged from nursing homes. The scope of this paper was to present the latest data regarding the COVID-19 spread in care homes worldwide, identifying causes and possible solutions that would limit the outbreaks in this overlooked category of population. It is the authors' hope that raising awareness on this matter would encourage more studies to be conducted, considering the fact that there is little information available on the impact of the SARS-CoV-2 pandemic on nursing homes. Establishing national databases that would register all nursing home residents and their health status would be of great help in the future not only for managing the ongoing pandemic but also for assessing the level of care that is needed in this particularly fragile setting.

## 1. Introduction

In December 2019, several cases of pneumonia of unknown origin were reported in Wuhan, China [1], leading to the identification of a new RNA coronavirus which was named SARS-CoV-2 (severe acute respiratory syndrome coronavirus 2). The nomenclature derived from its genetic similarities to other viruses from the Coronaviridae family, namely, over 80% similarity with SARS-CoV (severe acute respiratory syndrome coronavirus) and 50% with MERS-CoV (Middle East respiratory syndrome coronavirus), both responsible for two high-mortality epidemics in the last two decades [2–6]. The novel coronavirus has quickly spread from its point of origin to various continents, determining the World Health Organization (WHO) to declare the COVID-19 (coronavirus disease 2019) to be pandemic on March 11^th^, expressing their concerns regarding both the spread of the virus and the inaction of the authorities [7].

As of August 10^th^, 2020, a number of 19,718,030 cases have been reported worldwide, with a total of 728,013 deaths [8]. According to the WHO, some of the most affected countries were the United States of America, with 14,960 confirmed cases per 1 million population and 486 deaths per million, Peru with 14,285 confirmed cases and 632 deaths per 1 million population, and Brazil with 14,172 confirmed cases and 472 deaths per million [9]. The COVID-19 pandemic forced authorities in several countries to impose strict restrictions on the general population to limit the spread of the virus, leading to an economic backlash and a healthcare crisis.

Previous studies showed that the elderly and those with preexisting conditions, attributes that also describe most residents of long-term care facilities, had a significantly higher risk of severe disease and death. Nursing homes were possibly the most vulnerable institutions even before the COVID-19 pandemic, the CDC (Centers for Disease Control and Prevention), indicating in a study in 2014 that 80% of nonfood borne outbreaks of norovirus in the USA took place in long-term care facilities [10].

Since the beginning of the ongoing pandemic, numerous outbreaks of SARS-CoV-2 have been reported in nursing homes worldwide, affecting both the residents and the care workers. In the USA, it was estimated that 35,000 reported deaths due to COVID-19 emerged in nursing homes, representing 42% of the total number of COVID-19 deaths [11], with fatality rates amongst the residents reaching up to 33.7% in a care facility in King County, Washington [12]. Similar data regarding the percent of nursing home COVID-19 deaths in relation to the total COVID-19 deaths were also reported in numerous countries in Europe, such as Belgium (42%), France (44.6%), and Ireland (54%) [13]. It is crucial to identify and correct the possible shortcomings related to nursing home procedures to better manage the COVID-19 pandemic and any other future epidemics.

## 2. The High-Risk Characteristics of Nursing Home Residents

Since the beginning of the pandemic, researchers indicated that the aged population should be shielded from the virus, as they pose a higher risk of developing a more severe form of the disease, which could further lead to increased mortality [14]. Studies showed that age over 60-65 years was associated with a significantly higher risk of severe and critical manifestations, disease progression, and exitus, with men being more at risk for worse outcomes (see Table 1) [14–16]. One prospective, 12 months study conducted in 8 countries on 4156 residents of nursing homes found that the mean age of the residents was 83.4 ± 9.4 years, and that 81.3% of them suffered from ADL (activities of daily living) disability, while 68.0% presented cognitive impairment [17], thus representing one of the most at-risk population in light of the recent SARS-CoV-2 outbreak.

Comorbidities such as diabetes, cardiovascular disease, chronic respiratory disease, cerebrovascular disease, malignancy, and dementia were proved to independently increase the risk of COVID-19 progression, severe outcomes, and death (see Table 1) [15, 16, 18–23]. One previous study, conducted on 43,510 nursing home residents in the USA, showed that 65.5% were diagnosed with dementia, 46.8% had hypertension, 20.9% suffered a stroke, 20.6% had diabetes mellitus, and 14.3% chronic obstructive pulmonary disease, all of these preexisting conditions being associated with 1-year mortality more than 30%, even higher for the latter one which generated 1-year mortality of up to 43.8% [24]. Similar to the study presented above, this paper also found the mean age of the residents to be 84.4 ± 7.8 [24]. Considering the fact that most of the participants had more than one comorbidity, it is predictable that the impact of a SARS-CoV-2 infection would be severe. Thus, a strategy for protecting this category of population from contracting SARS-CoV-2 is imperative.

Another possible shortcoming that may contribute to the high infection rate amongst nursing homes is the fact that residents of long-term care facilities frequently suffer from various degrees of disability, and this may often lead to inability to properly perform preventive health measures, such as diligent hand washing. One study that focused on determining the level of disability amongst diabetes-diagnosed nursing home residents found that the mean number of underlying comorbidities was 4, the most frequent being dementia, stroke, hypertension, and ischemic heart disease. More than half of the residents (62%) were not able to feed themselves, 51% had speech impairment, and 89% required at least a frame or the help of one carer in order to walk [25]. Consequently, is therefore likely that the physical interaction between the facility personnel and these residents is prolonged, thus increasing the risk of contamination on both parts. Studies on previous epidemics also showed that the high number of outbreaks in nursing homes may also be explained by the large numbers of residents that share the same sources of water, food, air, the same facilities, and the fact that a limited number of care workers are responsible for numerous inhabitants [26, 27].

Implementing protocols that would address these matters, together with establishing training programs for all care workers might diminish the number and the severity of outbreaks that take place in nursing homes.

## 3. Impact on Nursing Homes

As was the case with many other natural disasters, such as hurricane Katrina, nursing homes have been one of the most affected sectors by the COVID-19 pandemic [28]. Studies that analysed previous influenza epidemics and the way they impacted long-term care homes made a number of recommendations on how to better manage the spread of a pathogen in these facilities [29–32]. In spite of considerable efforts being made to protect residents, the response to this healthcare crisis still needs to be improved to avoid further lives to be lost.

In addition, data regarding the residents, which should be easily accessible, is still lacking altogether with standardization of this data and cross-sector cooperation on how to gather, share, and make use of it, leading to a scarcity of basic information, such as the number of residents and deaths among them [33]. Due to these limitations encountered across most nations, there is not enough knowledge about the true impact of COVID-19 in this environment. However, a few countries managed to report relevant data, and several studies were conducted, which could partially shed light on the grim picture inside care homes around the world.

One of the countries with the most comprehensive data set is the United States of America. The Nursing Home COVID-19 Public File is a document that contains a set of information reported by the care homes to the CDC's National Healthcare Safety Network (NHSN). This includes updated details about the impact of the SARS-CoV-2 infection upon the residents, the capacity of each facility, and information about the personnel and protective equipment, as well as ventilator capacity. As of August 6th, 2020, the latest document comprised the data reported by 153,853 nursing homes across the USA and was showing the following numbers: 164,055 confirmed cases among residents, with an additional 102,531 suspected cases and 43,231 total COVID-19 deaths. The most affected states were California, Texas, Pennsylvania, New York, and New Jersey, which reported between 11,000 and 14,000 total confirmed cases each, the last three, and Massachusetts had the highest death toll (3,510-4,476). Furthermore, New Jersey, Connecticut, and Massachusetts top the list for the average number of cases per 1000 residents, with values of 359.2, 350.1, and 337.4, respectively. Massachusetts and New Jersey also show a disproportionately higher number of deaths per 1000 residents in comparison to the other states, with figures of 124.0 and 122.6. On the same day, the total number of cases in the USA was 5,034,140 with a total number of deaths of 162,807. This could mean that 3.26% of all cases in America were in nursing homes, while 26.55% of all deaths targeted the same group. However, the percentages may be underestimated, taking into consideration the fact that the report only started on 17th May 2020, and not all care homes are included. Moreover, some of the ones that are part of the report only showed the figures from the 17th May forward [34].

Another country that has attempted to look into the impact of the pandemic on the most vulnerable was the United Kingdom. The Office for National Statistics (ONS) conducted the Vivaldi study, a large-scale survey performed on nursing homes in England between the 26th May and 20th June 2020. Out of the total, 5,126 (56%) care homes responded. The study included approximately 9,081 home cares (95% CI: 293,168 to 293,434), with 293,301 residents and 441,498 staff members (95% CI: 441,240 to 441,756). More than half of the nursing homes (95% CI: 55%-56%) reported at least one confirmed case (amongst residents or staff members), with 20% (95% CI: 19% to 21%) of the residents, and 7% (95% CI: 6% to 8%) of the staff estimated to have tested positive for the SARS-CoV-2 infection [35].

In addition to this study, on the 3rd July 2020, the ONS also released a report which contained the number of deaths that occurred before the 12th June and were registered before the 20th June 2020 in England and Wales care sector: Out of 66,112 total deaths among the nursing homes residents, 19,394 (29.3%) were SARS-CoV-2 related [36].

Furthermore, in the year-to-date analysis for the week ending on the 31st July 2020, the ONS reported that 29.7% of Covid-19 deaths in England and Wales occurred in care homes, 4.7% in private homes, and 1.4% in hospices [37].

Perhaps the most comprehensive study performed so far in this field is the one conducted at the London School of Economics and Political Sciences. This was last updated on the 3rd of May and comprises the reports from 13 different countries around the world. The authors conclude that the nursing home share of COVID-19 deaths tends to be lower in the countries that have seen fewer fatalities in total. For example, according to their report, Hong Kong and Singapore had 0 and 2 deaths, respectively, in care homes, out of 4 and 18 deaths in total, in comparison to countries situated at the other extreme, which reported percentages of COVID-19-related deaths in their nursing homes between 19% in Hungary and 62% in Canada [38] (see Table 2).

- The first country considered by the study was Australia which registered, according to the paper, a total number of 95 Covid-19-related deaths on the 3rd May, out of which 24 were residents of subsidized aged care facilities, meaning that 25.3% of deaths were encountered in care homes [38]. However, on the 12th of August, the total number of COVID-19 deaths in Australia was 331, out of which 220 were residents of subsidized aged care facilities. This equates to a percentage of 66.5% [39].
- Another country that was included in the study and published the official number of Covid-19-related deaths was Belgium. On the 3rd of May, Belgium was reporting a total number of 7844 deaths involving SARS-CoV-2. Of these, 4164 fatalities occurred in care homes, leading to a percentage of 53.1% [40]. At the same time, the study shows an increase from 42% on the 11th of April, when Belgium first announced the official data regarding the number of deaths within care homes in the current pandemic. As of the 11th of August, the total number of COVID-19-related deaths in Belgium is 9,879, out of which 4,897 were care home residents (49.6%) [38].
- In Canada, the vast majority of COVID-19 cases were concentrated in Alberta, British Columbia, Quebec, and Ontario. On the 2nd of May, the Public Health Agency of Canada reported a total of 3,566 deaths, with 2,227 of these affecting nursing home residents, which equals to 62% of the total number of deaths [40]. However, on the 25th of May, the Canadian Institute for Health Information released a document that found a proportion of 81% of novel coronavirus-related fatalities occurring in long-term care facilities [41]. On August 11th, Canada registered a total of 8,991 deaths, out of which 7,028 were in care homes, translating into a percentage of 78.2% [42].
- On the 2nd May 2020, the French Ministry of Health reported a total number of 24,760 COVID-19-related deaths. According to the report, 51% of cases (12,511 patients) occurred among care home residents: 9,273 of the patients deceased in the care facilities and were mainly “probably cases,” and 3,238 deceased in hospitals (laboratory-confirmed cases of Covid-19) [40]. Subsequently, on the 3rd of August, figures showed that out of a total number of 30,294 deaths, 10,420 nursing home residents died in the establishments and 3,696 died in hospitals, which correlates with a percentage of 46.6% [43].
- Germany's Robert Koch Institute reported on the 3rd May that out of 6,649 total Covid-19-related deaths, 2,401 affected residents living in communal settings. This resulted in a 36.1% share, which was lower than in other countries with a similar number of deaths [40]. It is essential to point out that the database of the abovementioned German Institute includes not only nursing homes but also other additional communal settings that cover a wide range of health and social issues (such as homeless shelters, facilities for asylum-seekers, or other mass accommodation). On the 11th of August, the total number of deaths was 9,201, and the share in communal settings increased to 3,641 (39.6% of deaths) [44].
- In Italy, the National Institute of Health released on the 6th April the results of a survey conducted on 2,166 out of Italy's 4,629 care homes. The study showed a number of 3,859 total deaths in the nursing homes that responded between the 1st of February and the 6th of April, 37% of these being linked to the SARS-CoV-2 infection [40].
- On April 3rd, the Spanish Health Ministry required the homogenous gathering and reporting of data regarding the situation in nursing homes from every regional government. Official information has never been released before May 3rd; however, according to the National Television of Spain, which published data regularly, 16,878 COVID-19-related deaths occurred in care homes, adding up to 67.0% of the total number in Spain at that time [40]. The latest figures show that on the 10th of August, 19,664 people were reported to have deceased from SARS-CoV-2-related illness in care homes, which represents the equivalent of 68.8% of total COVID-19-related fatalities

## 4. Perspectives and Recommendations

During the COVID-19 pandemic, care home residents found themselves to be the most vulnerable population and probably amongst the least sheltered individuals. Part of the reasons that led to dramatic outcomes inside the nursing home facilities was a lack of official guides and regulations in case of natural disasters for elderly shelters around the world. Following the novel coronavirus global outbreak, some of the mechanisms responsible for issuing such instructions released a set of guidelines for this particular purpose.

The body responsible for setting this type of regulations across the European Union is called the European Centre for Disease Prevention and Control (ECDC). On May 19th, 2020, ECDC released a technical report regarding the surveillance of COVID-19 in the EU/EEA nursing homes [46]. The paper outlines the major points to be addressed in long-term care facilities, to avoid a further underestimation of the disease burden upon these settings. Members of staff working while contagious (symptomatic or asymptomatic carriers of SARS-CoV-2) represented one of the leading causes of an increased COVID-19 spread inside care home facilities. Other promoting factors were represented by staff under qualification or inadequately training, working in multiple facilities, shortage of personal protective equipment, or limited testing performed only on symptomatic individuals. Furthermore, the increased incidence of neurological pathologies (namely dementia) that commonly affect residents of care homes can lead to a more difficult diagnosis.

The ECDC advises the implementation of a screening system for residents (consisting of daily monitoring for symptoms and periodical testing), and that the staff members who care for residents should be tested on a regular basis (e.g., weekly). Whenever a confirmed COVID-19 case is identified, appropriate infection prevention and control measures should be enabled promptly. Facility-based data should be collected by every care home, preferentially with the aid of an integrated electronic system for tracking and monitoring the health status of both residents and staff members. Notification to the relevant health authorities is essential; hence, data should be reported daily to the local, regional, national, and EU designated mechanisms. The ECDC also highlights the importance of nonpharmaceutical interventions, such as face mask usage, isolation of sick individuals, and strict adherence to hygiene guidelines [46].

Another set of guidelines was released by the Centers for Disease Control and Prevention in the United States of America, on the 30^th^ of April 2020. The recommendations include the establishment of a designated COVID-19 care unit for the confirmed cases among the residents. This could be a different wing, floor, or small group of rooms, according to the capacity of each facility. Additionally, the CDC advises that new and readmitted nursing home residents with confirmed COVID-19, that are still required to undergo transmission-based precautions, should also be placed in the COVID-19 care unit. Further, measures discuss the isolation of suspected cases or the contacts of confirmed ones, with further testing prioritization. The latter also advises for offering priority to symptomatic staff or residents in case of limited testing capacity [47].

## 5. Conclusions

The Covid-19 pandemic has affected the vulnerable people in care homes more than any other category, with percentages between 19% and 72% of all SARS-CoV-2-related deaths occurring amongst these facilities. Once again, the health and social care sectors have proven their interdependence, when the shortages encountered in healthcare became obstacles in sheltering and providing for the most fragile of our kind. A lack of standardized guidelines, discrepancies between public and private sectors, underfunding, and many other well-established irregularities have led to a dramatic outcome for the nursing home residents. The hope is that unfortunate events that could have been prevented will lead to a decisive shift in policies regarding these facilities and the avoidance of future similar outcomes.

## Figures and Tables

**Table 1:** Risk factors for severe disease and death in COVID-19 patients—a summary from multiple recent studies.

		Study					
Risk factors for severe disease and death		Zheng et al. [19]	Li et al. [15]	Zhang et al.^∗^ [16]	Wang et al. [21]	Bianchetti et al. [23]	Docherty et al.^∗∗^ [20]
	Age over 65 years	OR = 6.06, 95% CI (3.98-9.22)	OR = 2.2, 95% CI (1.5-3.5)	OR = 4.791, 95% CI (3.018-7.606)			
	Male gender	OR = 1.76, 95% CI (1.41-2.18)		OR = 0.520, 95% CI (0.355-0.761)			
	Smoking	OR = 2.51, 95% CI (398-9.22)					
	Diabetes	OR = 3.68, 95% CI (2.68-5.03)			OR = 2.47, 95% CI (1.67-3.66)		
	Hypertension	OR = 2.72, 95% CI (1.60-4.64)	OR = 2.0, 95% CI (1.3-3.2)		OR = 2.29, 95% CI (1.69-3.10)		
	Cardiovascular disease	OR = 5.19, 95% CI (3.25-8.29)		OR = 2.436, 95% CI (1.503-3.948)	OR = 2.93, 95% CI (1.73-4.96)		HR = 1.16 (1.08-1.24)
	Respiratory disease	OR = 5.15, 95% CI (2.51-10.57)			OR = 5.97, 95% CI (2.49-14.29)		HR = 1.17 (1.09-1.27)
	Cerebrovascular disease				OR = 3.89, 95% CI (1.64-9.22)		HR = 1.17 (1.06-1.29)
	Dementia					OR = 1.84, 95% CI (1.09-3.13)	HR = 1.40 (1.28-1.52)
	Malignancy						HR = 1.13 (1.02-1.24)

^∗^Only patients over 60 years old were taken into consideration. ^∗∗^The HR values are calculated for the hazard representing death. All the values presented in the table had statistical significance (*p* value <0.05).

**Table 2:** Number of COVID-19-related deaths in care homes in May and August 2020.

Country	April/May deaths				August deaths			
	Total population	Care homes	% in care homes	Reference	Total population	Care homes	% in care homes	Reference
Australia	95	24	25.3%	[40]	331	220	66.5%	[39]
Belgium	7844	4164	53.1%	[40]	9879	4897	49.6%	[38].
Canada	3566	2227	62.5%	[40]	8991	7028	78.2%	[42]
France	24760	12511	50.5%	[40]	30294	10420	34.4%	[43]
Germany	6649	2401	36.1%	[40]	9201	3641	39.6%	[44]
Spain	25191	16878	67.0%	[40]	28581	19664	68.8%	[45]
England and Wales	22351	4158	18.6%	[40]	51710	15334	29.7%	[36]
United States	—	—	—		162807	43231	26.6%	[34]
